# RNAi-Mediated Functional Analysis of Bursicon Genes Related to Adult Cuticle Formation and Tanning in the Honeybee, *Apis mellifera*

**DOI:** 10.1371/journal.pone.0167421

**Published:** 2016-12-01

**Authors:** Claudinéia Pereira Costa, Moysés Elias-Neto, Tiago Falcon, Rodrigo Pires Dallacqua, Juliana Ramos Martins, Marcia Maria Gentile Bitondi

**Affiliations:** 1 Departamento de Genética; Faculdade de Medicina de Ribeirão Preto; Universidade de São Paulo; Ribeirão Preto, SP, Brazil; 2 Departamento de Biologia; Faculdade de Filosofia, Ciências e Letras de Ribeirão Preto; Universidade de São Paulo; Ribeirão Preto, SP, Brazil; 3 Centro de Ciências Biológicas e da Saúde; Universidade Federal de Mato Grosso do Sul; Campo Grande, MS, Brazil; CNRS, FRANCE

## Abstract

Bursicon is a heterodimeric neurohormone that acts through a G protein-coupled receptor named rickets (rk), thus inducing an increase in cAMP and the activation of tyrosine hydroxylase, the rate-limiting enzyme in the cuticular tanning pathway. In insects, the role of bursicon in the post-ecdysial tanning of the adult cuticle and wing expansion is well characterized. Here we investigated the roles of the genes encoding the bursicon subunits during the adult cuticle development in the honeybee, *Apis mellifera*. RNAi-mediated knockdown of *AmBurs α* and *AmBurs β* bursicon genes prevented the complete formation and tanning (melanization/sclerotization) of the adult cuticle. A thinner, much less tanned cuticle was produced, and ecdysis toward adult stage was impaired. Consistent with these results, the knockdown of bursicon transcripts also interfered in the expression of genes encoding its receptor, AmRk, structural cuticular proteins, and enzymes in the melanization/sclerotization pathway, thus evidencing roles for bursicon in adult cuticle formation and tanning. Moreover, the expression of *AmBurs α*, *AmBurs β* and *AmRk* is contingent on the declining ecdysteroid titer that triggers the onset of adult cuticle synthesis and deposition. The search for transcripts of *AmBurs α*, *AmBurs β* and candidate targets in RNA-seq libraries prepared with brains and integuments strengthened our data on transcript quantification through RT-qPCR. Together, our results support our premise that bursicon has roles in adult cuticle formation and tanning, and are in agreement with other recent studies pointing for roles during the pharate-adult stage, in addition to the classical post-ecdysial ones.

## Introduction

The rigid cuticle covering the insects is periodically renewed during the discontinuous body growth. Such renewal events, or molts, start with the detachment of the actual cuticle from the epidermis (apolysis) and is followed by the formation of a new cuticle beneath the detached one. Molting periodicity is regulated by the fluctuation of ecdysteroid hormones. A peak in the titer of ecdysteroids triggers the apolysis and marks the beginning of a molting episode. The single-layered epidermis then synthesizes the new cuticle as the hormone titer gradually decreases toward basal levels. The next event is the ecdysis when the old cuticle is shed thus exposing the quasi-completely renewed cuticle [1–3]

Proteins and the polysaccharide chitin are the main raw materials forming the cuticle. The cuticular proteins may bear diagnostic domains, such as the R&R Consensus, typical of the CPR proteins, which has been identified as a chitin-binding domain [4–5]. In the absence of a chitin-binding Consensus, other sequence motifs have been characterized and used to identify and annotate cuticular proteins in sequenced genomes of insects and other arthropods. These sequence features have allowed the categorization of twelve classes of cuticular proteins, seven of them being represented in the *Apis mellifera* genome. The CPR family is by far the largest cuticular protein family in every insect species investigated until now [6]. Another cuticular protein family is Tweedle, first identified in a *Drosophila melanogaster* mutant [7] and with members widespread among the insects [6]. The *A*. *mellifera* genome contains two genes encoding Tweedle proteins [8, 9]. Members of this protein class share four conserved blocks of amino acids that have been considered as a characteristic signature [7]. Although lacking the R&R Consensus, it was demonstrated in *Bombyx mori* that Tweedle proteins bind chitin [10]. The apidermin family has been found exclusively in hymenopterans, specifically in *A*. *mellifera* [9–11], *Nasonia* wasps [6–12], and *Bombus terrestris* and *impatiens* [13]. Apidermins are highly hydrophobic, with a high percentage of alanine residues. Transcripts for three genes encoding apidermins were identified in the epidermis, trachea and digestive tract of *A*. *mellifera*; their expression patterns are consistent with roles of their respective proteins in cuticle structure. In *A*. *mellifera*, members of the CPR, Tweedle, and Apidermin protein families have been most studied regarding developmental regulation of gene expression in tissues and organs [8, 9, 11, 14]. The other cuticular protein families found in the honeybee are known by the abbreviations CPF, CPFL, CPLCP, and CPAP3 [6, 9]. Although a function as structural cuticle protein has been assigned to each of these protein classes, such diversity suggests that they display distinct, and yet unknown, functions in the cuticle.

The formation of the adult cuticle, or definitive exoskeleton, in the honeybee, culminates in a series of chemical reactions leading to melanization and sclerotization, or tanning. Such attributes turn the exoskeleton hard and dark and are required for its complete functionality. The biochemical pathway leading to cuticle tanning involves tyrosine hydroxylation by a tyrosine hydroxylase to obtain 3,4-dihydroxyphenylalanine (dopa), decarboxylation of dopa by dopa decarboxylase to form dopamine, which serves as a precursor for the synthesis of melanin and also forms N-acetyldopamine and N-β-alanyldopamine. Both of these catechols are precursors of quinones that can react with amino acid residues in the cuticular proteins and also with chitin, thus forming crosslinks and stabilizing the cuticle [15]. In addition to the enzymes cited above, peroxidases have also been pointed as potential catalysts in oxidative reactions leading to cuticle tanning and protein cross-linking [15, 16–18].

In this context, the neurohormone bursicon, a product of neurosecretory cells in the central nervous system, has been considered an essential player in the post-ecdysial (post-eclosion) events related to adult cuticle tanning and wing expansion in insects [19–26]. Bursicon is a heterodimer consisting of two cystine knot polypeptides, Burs α and Burs β, which is released into the hemolymph. As verified in *Drosophila*, bursicon stimulates the increase in intracellular levels of cAMP via interaction with its receptor, a leucine-rich repeats-containing G protein-coupled receptor 2 (DLGR2), which is encoded by the gene rickets (rk) [27–30]. cAMP acts by activating a protein kinase A, which phosphorylate tyrosine hydroxylase [31], the rate-limiting enzyme in the melanization/sclerotization pathway.

Bursicon is the last hormone in the neuropeptide signaling cascade triggered by the decline in the ecdysteroid titer following apolysis, and ultimately leading to adult ecdysis and post-ecdysial events. The cascade starts with the release of the ecdysis-triggering hormone that induces an increase in eclosion hormone levels, which in turn causes the release of the crustacean cardioactive peptide and bursicon [31]. The post-ecdysial roles of bursicon in adult cuticle tanning and wing inflation are well characterized [19–26]. The aim of the current study was to search for evidence supporting a role of bursicon during the period encompassing the pharate-adult development culminating in the adult ecdysis. During this time interval, the adult cuticle is synthesized and differentiates as a dark and hard structure. Bursicon may play roles in this tanning process. To shed light on this issue, we characterized the expression of the genes encoding the bursicon subunits during this time interval, and in relation to the ecdysteroid titer that coordinates adult cuticle synthesis. By interfering in the levels of bursicon transcripts through RNAi, we could investigate its regulatory action on candidate target genes encoding structural cuticular proteins and enzymes in the tanning pathway, and on the gene encoding its putative receptor.

## Material and Methods

### Honeybees

Africanized *A*. *mellifera* workers at different stages of development were obtained from hives maintained at the Experimental Apiary of the Universidade de São Paulo, Ribeirão Preto, SP, Brazil. Pupae and pharate adults were staged according to the criteria established by Thompson [32] and Michelette and Soares [33]. Adults were collected as they emerged from brood cells (0 h newly-ecdysed bees).

### Genes encoding bursicon, its receptor and putative targets

The assembled honeybee genome (versions 4.0 and 4.5) was searched using the *burs α*, *burs β* [29] and *rk* [34] gene sequences of *D*. *melanogaster* as query in BLASTN and BLASTP analyses. Predicted honeybee genes, herein named *AmBurs α* and *AmBurs β*, were identified as the best candidates for encoding the honeybee subunits of bursicon, AmBurs α and AmBurs β. The gene *AmRk* was identified as encoding the bursicon receptor, AmRk. In parallel, genes acting in the integument, whose expression patterns were previously characterized in our laboratory, were herein investigated as potential targets of bursicon. These genes encode two cuticular proteins from the CPR family, *AmCPR14* [14] and *AmCPR3* [9], two Tweedle proteins, *AmTwdl1* and *AmTwdl2* [8], two apidermins, *Amapd2* and *Amapd3* [9, 11], and three enzymes potentially acting in the cuticular melanization/sclerotization pathway, namely, tyrosine hydroxylase (*Amth*) and dopa decarboxylase (*Amddc*) and a peroxidase (*Ampxd*) [8]. The accession numbers for all these genes are given in S1 Table.

### cDNA synthesis and amplification

Total RNA was extracted from brains and integuments of pupae, pharate adults and newly ecdysed adults using the Trizol protocol (Invitrogen). The samples were incubated with RNase-free DNase I (Invitrogen) to eliminate contaminant DNA. First strand cDNA was synthesized from a standard amount of total RNA through reverse transcription using SuperScript II reverse transcriptase (Invitrogen) and an oligo (dT)_12-18_ primer (Invitrogen). Negative control reactions without the enzyme were prepared in parallel to check for genomic DNA contamination. cDNA samples were amplified through semiquantitative PCR (sqPCR) or quantitative PCR (qPCR). The reaction mixtures for the sqPCR were prepared with 0.8 μl (10 μM) of the specific primers (*AmBurs α* and *AmBurs β*, S2 Table), 1 μl cDNA and 10 μl Eppendorf Master Mix 2.5x in a total volume of 20 μl in water. The gene encoding a ribosomal protein, *AmRP49*, which is expressed in similar levels during honeybee development, and was validated as being suitable for normalizing PCR data [35] was used to control cDNA loading and to correct for differences in cDNA amounts. The primers used for the targets and reference genes (S2 Table) were designed to span at least an intron, thereby serving as control for genomic DNA contamination. The sqPCR conditions for the target genes were 95°C for 2 min, 30 cycles (desnaturation/anneling/extension) of 94°C for 30 sec, 60°C for 45 sec, 72°C for 50 sec and 72°C for 10 min. The same conditions, except the number of cycles (27 cycles), were used for the *AmRP49* reference gene amplification. The PCR product was analyzed through electrophoresis in 1% agarose gels in 1x TBE (0.45M Tris Base, 0.45M boric acid, 0.5M EDTA, pH 8.0) containing ethidium bromide. A 100 bp molecular weight marker was added to one of the gel lanes. After electrophoresis, the gels were examined with a Hoefer MacroVue UV-20 transilluminator coupled with a Kodak Edas 290 equipment.

The relative quantification of transcripts (qPCR) was carried out in a 7500 Real Time PCR System (Applied Biosystems) using 20 μl of a reaction mixture containing 10 μl SYBR Green Master Mix 2x (Applied Biosystems), 1 μl cDNA, 7.4 μl water, and 0.8 μl of each gene-specific primer (*AmBurs α*, *AmBurs β*, *AmRk*, *AmCPR14*, *AmCPR3*, *AmTwdl1*, *AmTwdl2*, *Amapd2*, *Amapd3*, *Amddc*, *Amth* and *Ampxd*). The *AmRP49* gene was used as the endogenous reference. All primers (S2 Table) were designed to span at least one intron. Reactions not including the cDNA template were prepared as negative controls. The PCR conditions were 50°C for 2 min and 95°C for 10 min followed by 40 cycles of 94°C for 1 min, 60°C for 30 s and 72°C for 30s. Each run was followed by a melting curve analysis to confirm the specificity of amplification and absence of primer dimers. In order to check reproducibility, each SYBR green assay was carried out in technical duplicates or triplicates and repeated with three or four independent biological samples. The baseline and threshold were correctly set to obtain accurate Ct values, which were exported into a MS Excel spreadsheet (Microsoft Inc) for 2^-∆∆C^_T_ calculation (Applied Biosystems User bulletin #2; [36]).

### Cloning, sequencing, alignment and phylogenetic analysis

Using the specific primers for *AmBurs α*, *AmBurs β* and *AmRk* (S2 Table), cDNA regions were amplified and electrophorized in 1% agarose gels containing ethidium bromide. The PCR products were recovered and purified with MinElute PCR Purification Kit (Qiagen). The purified *AmRk* PCR product was directly sequenced and the purified *AmBurs α* and *AmBurs β* PCR products were cloned using pGEM-T Easy Vector kit (Promega). After ligation into the plasmids and transformation of DH5α competent cells, the bacteria were grown in LB medium in the presence of X-Gal, ampicillin and IPTG. Insert-containing plasmids (white colonies) were isolated using FastPlasmid Mini (Eppendorf). The presence of the insert was confirmed by digesting plasmids with EcoRI (Invitrogen). Dideoxy sequencing of *AmBurs α*, *AmBurs β* and *AmRk* was performed in an ABI Prism 310 DNA Analyzer (Applied Biosystem) using BigDye Terminator v3.0 Cycle Sequencing Ready Reaction (Applied Biosystems) and M13 forward and reverse universal primers for *AmBurs α* and *AmBurs β*, and specific primers for *AmRk* (S2 Table). The obtained nucleotide sequences were analyzed using Sequencher (version 4.7) for Windows (Gene Codes Corporation) and compared against the *A*. *mellifera* annotated genome (versions 4.0 and 4.5) using the Artemis 7.0 platform [37]. The complete consensus sequences of *AmBurs α* and *AmBurs β* was submitted to the National Center for Biotechnology Information's (NCBI) GenBank (http://www.ncbi.nlm.nih.gov).

AmBurs α, AmBurs β and AmRk amino acid sequences were aligned with orthologue sequences from other arthropod species available at the NCBI. For sequence IDs, see S3 Table. The alignments were performed using the online version of the software MAFFT v. 7 [38] (http://mafft.cbrc.jp/alignment/server/) in default standard and visualized in the ClustalX v.2.1 graphical alignment software [39]. Then, we performed a model test for the resultant Burs α and Burs β subunits aligned files using the software ProtTest v. 3.2 [40] with default parameters to find the best amino acids substitution model. The indicated model for Burs α was JTT [41] with an estimative of gamma heterogeneity rate between sites (+G), and for Burs β was LG [42], with an estimative of invariable sites (+I) and +G. The -LnL (log likelihood) values were 3141.88 for Burs α and 3763.48 for Burs β. We used the parameters indicated by the model tests in phylogenetic analyses through the software PhyML v. 3.1 [43]. A maximum likelihood analysis [44] was performed with 1000 bootstrap replications for each Burs subunit. Model test and phylogenetic analysis for rickets were not performed due to the scarcity of available sequences.

### RNA-seq libraries preparation

Pharate adults (Pbm phase) and newly-ecdysed adult bees (NE phase) were dissected for the collection of brain and integument samples. Two brain samples (one per developmental phase) were prepared, each containing five brains. Six integument samples (three per developmental phase) were also prepared, each containing five integuments. Total RNA was extracted from these eight samples using the Trizol protocol. The extracted RNA was sent to a facility (Laboratório Central de Tecnologias de Alto Desempenho—LaCTAD, Campinas, SP, Brazil) for preparation of the RNA-seq libraries (TruSeq^TM^ RNA Sample Preparation, 2x100 bp) and sequencing on an Illumina HiSeq 2500 equipment. After sequencing, we trimmed the adapters' sequences using the software Cutadapt v. 1.4.1 (http://code.google.com/p/cutadapt/) [45] and Scythe v. 0.991 (https://github.com/vsbuffalo/scythe). The trimmed sequences were evaluated with the software FastQC v. 0.11.2 [46]. The high-quality reads were aligned against the *A*. *mellifera* genome v. 4.5 through the software TopHat v. 2.0.9 (http://tophat.cbcb.umd.edu) [47]. We used the software Cufflinks (http://cufflinks.cbcb.umd.edu) [48] to evaluate the assembly. The package Cuffmerge integrated the mapped reads, and the package Cuffdiff indicated the transcript expression levels for each sample. We extracted the information for the transcripts of interest (*AmBurs α*, *AmBurs β*, *AmRk*, *AmCPR14*, *AmCPR3*, *AmTwdl1*, *AmTwdl2*, *Amapd2*, *Amapd3*, *Amddc*, *Amth* and *Ampxd*) using the R (v. 3.1.2) package CummeRbund v. 2.8.2 (http://compbio.mit.edu/cummeRbund/).

### Treatment with 20-hydroxyecdysone (20E)

The hormone 20E was first diluted in absolute ethanol (Merck) and then in Ringer solution (5 g NaCl, 0.42 g KCl, 0.25 g CaCl_2_.2H_2_O in 100ml H_2_O) (1:5 v/v) to obtain a final concentration of 2.5 μg/μl. A volume of 2 μl of this solution, making 5 μg, was injected into the hemocoel of pupae (Pw), in the abdome, using a Hamilton microsyringe (1701LT) and a G30 needle (Becton Dickinson). Controls were injected with vehicle only (2 μl of ethanol in Ringer, 1:5 v/v). The injected pupae were maintained in an incubator at 34°C and 80% relative humidity. Brain and integument samples were collected from experimental and control groups when controls reached the Pb and Pbd pharate-adult phases. The samples were homogenized in Trizol reagent and stored at -80°C for subsequent RNA extraction. So as to assess the effects of 20E, we performed RT-qPCR analysis with the primers listed in S2 Table for quantification of the abundance of transcripts encoding AmBurs α and AmBurs β bursicon subunits, and the specific receptor, AmRk.

### Knockdown of *AmBurs α* and *AmBurs β*

Specific primers were designed for *AmBurs α* and *AmBurs β* genes using Primer3 (http://frodo.wi.mit.edu/primer3/). Each primer was added to the T7 promoter sequence (S2 Table). The respective dsRNAs comprising 159 and 140 bp were synthesized by *in vitro* transcription with Ribo Max^TM^ Large Scale RNA Production Systems-T7 kit (Promega), using pupae and pharate adults brain pools (7 brains). The same procedure, but using a pool of larvae at the 3^rd^, 4^th^, and 5^th^ instars, and primers for a gene encoding a hexamerin, *AmHex 70b* (S2 Table), was used for the synthesis of a 69 bp dsRNA. *AmHex 70b* is highly expressed in the larval fat body [49] and served as a control for the dsRNA experiments. Each fragment was amplified by PCR under the following conditions: initial denaturation at 95°C for two minutes followed by 40 cycles at 94°C for 30 seconds, 60°C for 45 seconds, 72°C for 50 seconds, and a final extension at 72°C for 10 minutes. The reaction products were purified using the Wizard^®^SV Gel and PCR Clean-UP System kit (Promega). The synthesized dsRNAs were extracted with Trizol, re-suspended in nuclease-free water, heated at 95°C for 1 min and cooled.

Three groups of pupae (Pw phase) were injected with 2 μl of *AmBurs α* dsRNA (ds*AmBurs α*) or *AmBurs β* dsRNA (ds*AmBurs β*) at the final concentrations of 0.3, 1 or 3 μg in nuclease-free water. A fourth group was injected with nuclease-free water only (controls). Each group was formed by 15–20 pupae. The injection was intrabdominal and was made with the assistance of a Hamilton microsyringe coupled to a G30 needle (Becton Dickinson). After injection, the bees were maintained in an incubator at 34°C and 80% relative humidity for monitoring pharate-adult development and ecdysis to the adult stage. At the day of the ecdysis of the control bees, the total RNA was extracted from the whole body of individual bees (treated with ds*AmBurs α* or ds*AmBus β*, and controls) and used in RT-sqPCR analysis. In parallel, integuments of these bees were prepared for light microscopy after being dissected from the dorsal-medial region of the thorax, and from the medial region of the third abdominal tergite. At least three specimens of each group (treated with ds*AmBurs α*- or ds*AmBus β*, and controls) were dissected for histological sections. After a brief rinse in Ringer saline, the integuments were kept for 24 h in cold (4°C) fixative (4% paraformaldehyde in 0.1 M phosphate buffer, pH 7.3). The integuments were dehydrated in a graded ethanol series and then infiltrated for 24 h and embedded in methacrylate resin (Historesin, Leica). Sections of 5 μm were stained with methylene blue and basic fuchsin for 3 min, followed by a rapid rinse with distilled water. Sections were then mounted in Entellan (Merck) and examined and photographed using an Axioskop II photomicroscope (Zeiss).

Other four groups of pupae (Pw phase) were also established, each group containing 20 pupae. Three of these groups were respectively injected with 2 μl (2 μg) of ds*AmBurs α*, ds*AmBurs β* and ds*AmHex 70b* in nuclease-free water. The fourth group was left untreated. Since it is known that certain honeybee tissues are not easily reached by the dsRNA when it is injected into the abdominal cavity [50], the dsRNAs were delivered directly to the brain, in order to ensure penetration into the central nervous system, the local of *AmBurs α* and *AmBurs β* expression. All the three groups of pupae were placed in the incubator at the same conditions mentioned above. Brain and integument (thoracic and abdominal) samples were collected when controls reached the Pbl and NE phases, which are marked by the beginning of cuticle pigmentation and ecdysis, respectively. Total RNA samples extracted from brains and integuments were used in RT-sqPCR and RT-qPCR analyses with primers for *AmBurs α* and *AmBurs β* (brain samples) and primers for the genes encoding the structural cuticular proteins, enzymes of the melanization/sclerotization pathway and bursicon receptor (integument samples).

## Results

### Expression of the genes encoding bursicon and its receptor during the pharate-adult stage

**Fig 1:**
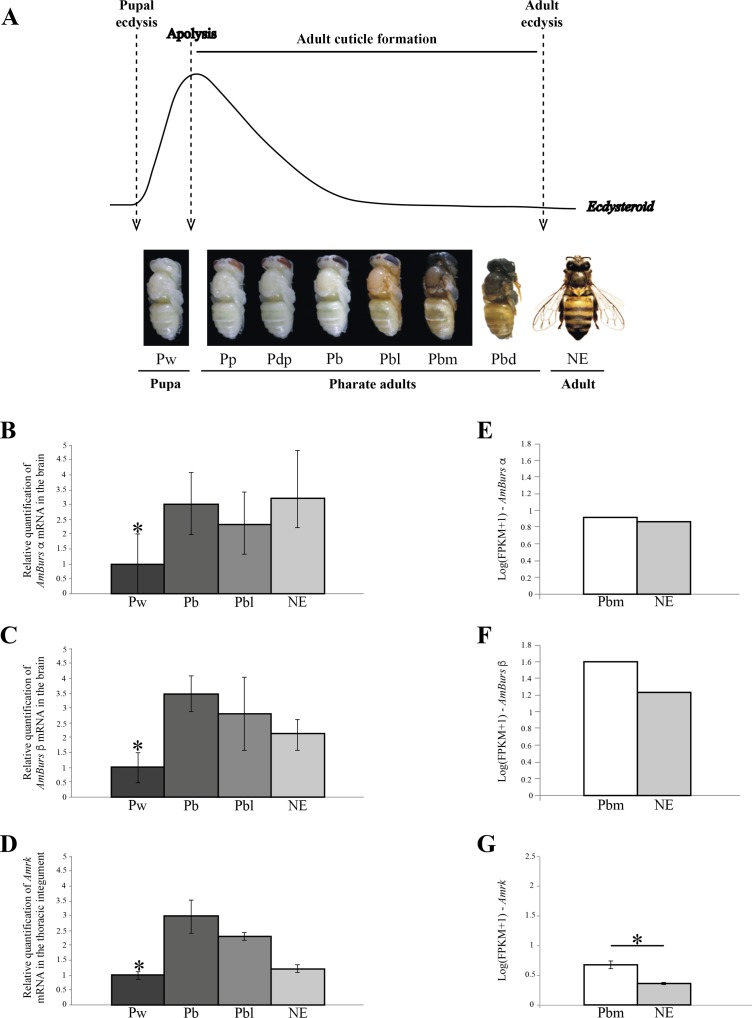
Expression of *AmBurs α and AmBurs β* in the brain, and expression of *AmRk* in the integument during adult cuticle formation. **(A)** The main molting events (pupal ecdysis, apolysis, synthesis and differentiation of the adult cuticle, adult ecdysis) are indicated in relation to the ecdysteroid titer variation and adult cuticle phenotype. Pupa (Pw), pharate-adults (Pp, Pdp, Pb, Pbl, Pbm and Pbd) and newly-emerged adults (NE) are the successive phases of the honeybee development. Relative quantification of **(B)** *AmBurs α* and **(C)** *AmBurs β* mRNAs in the brain of pupae (Pw), pharate adults (Pb, Pbl) and newly emerged-adults (NE). **(D)** Relative quantification of bursicon receptor (*AmRk*) transcripts in the integument of the same developmental phases. Transcript levels determined through RT-qPCR. Bars represent mean ± standard error (se) of three samples, each prepared with seven brains or seven thoracic/abdominal integuments. The asterisk indicates statistically significant increase of transcripts in pharate adults in relation to the pupal (Pw) phase (Student's t-test, p<0.05). **(E-G)** mRNA levels (FPKM+1) in the brain **(E, F)** and in the integument **(G)** of pharate adults (Pbm phase) and newly-ecdysed adults (NE phase) determined through RNA-seq. Levels of *AmBurs α* and *AmBurs β* were identified in two brain libraries, each prepared with five pooled brains extracted from the Pbm or NE phases. Levels of *AmRk* were identified in six integument libraries (three from Pbm and three from NE phases), each prepared with five integuments. The asterisk in **G** indicates a statistically significant difference (adjusted p value = 0.001).

The expression of the genes encoding bursicon, *AmBurs α* and *AmBurs β*, and its receptor, *AmRk*, was quantified in honeybee workers during the developmental interval between pupal and adult ecdyses. Such interval is marked by the synthesis of the adult cuticle, a process that initiates immediately after the ecdysteroid peak triggers the pupal cuticle apolysis (Fig 1A). The synthesis of the adult cuticle is followed by its maturation, which is marked by the onset of pigment deposition and intense sclerotization. Cuticular pigmentation starts in the Pbl phase and rapidly increases during the intermediate and final pharate adult phases (Pbm and Pbd) and in the newly-emerged bees (Fig 1A). In this context, we aimed to characterize whether the process of adult cuticle formation is dependent on bursicon gene expression. We chose four developmental phases within the pupal-to-adult ecdyses interval for quantifying the levels of bursicon transcripts in the brain and of its receptor, rickets (*AmRk*), in the integument. Our RT-qPCR data showed that the levels of *AmBurs α* (Fig 1B) and *AmBurs β* (Fig 1C) transcripts were significantly higher in the brains of pharate-adults and newly-emerged bees when compared to pupae, thus suggesting a role in adult cuticle formation. In support for such suggestion, the profile pattern of bursicon receptor transcripts in the integument followed the profile patterns of bursicon RNAs (Fig 1D).

We also searched for *AmBurs α* and *AmBurs β* transcripts in two RNA-seq libraries prepared with pooled brains from bees in process of adult cuticle formation (pharate-adults at the Pbm phase) and at ecdysis (NE phase). In parallel, we searched for transcripts of the bursicon receptor, *AmRk*, in six RNA-seq libraries prepared with integuments from the Pbm phase (three libraries) and from the NE phase (three libraries). Similar levels of *AmBurs α* and apparently similar levels of *AmBurs β* were found in the brains of pharate-adults (Pbm phase) (Fig 1E) and newly-ecdysed bees (NE phase) (Fig 1F), suggesting that ecdysis is not associated to significant changes in the levels of bursicon. The expression of *AmRk* was also detected at these same developmental phases, however, it was significantly higher in the integument of the pharate-adults (Pbm phase) (Fig 1G).

### Structure of the genes encoding bursicon and its receptor in the honeybee and similarity with putative orthologues in other arthropod species

In parallel with the respective transcript levels determination, *AmBurs α*, *AmBurs β* and *AmRk* cDNAs were sequenced and compared against the corresponding annotated genes in the honeybee genome assembly, thus allowing us to characterize the structure of these genes. *AmBurs α* and *AmBurs β* coding sequences span 462 and 438 nucleotides (stop codon included), distributed among four and three exons, respectively. Only the ten last (C-terminal) *AmBurs α* nucleotides and the fifteen last *AmBurs β* nucleotides were not validated by cDNA sequencing. Their conceptual subunits consist of 153 and 145 amino acids, have molecular masses of 16.9 and 16.6 kDa, and pI values of 7.96 and 4.99, respectively (http://web.expasy.org/protparam/). The respective N-terminal signal peptides span 22 and 28 amino acids. The search for conserved domains using BLAST Conserved Domain Database (http://www.ncbi.nlm.nih.gov/Structure/cdd/wrpsb.cgi) led to the identification of the following domains in the AmBurs α sequence: a C-Terminal Cystine Knot-like domain (CTCK), a Glycoprotein Hormone Beta domain (GHB) and the DAN domain, all of them typical of the CKP protein family. The DAN domain contains nine conserved cysteine residues able to form disulfide bonds. These domains were not found in the AmBurs β subunit (S1A–S1D Fig).

A potential orthologue of the *Drosophila* bursicon receptor gene, *rk*, had been previously identified in the *A*. *mellifera* genome and named *Am 47* [34]. This gene was herein renamed *AmRk*. The *AmRk* coding sequence spans 2991 nucleotides and has 13 exons. *AmRk* cDNA sequencing was limited to the interprimers region spanning 223 nucleotides. The predicted translation product consists of 951 amino acids; the signal peptide encompasses 23 amino acids. The calculated molecular mass is 150 kDa and pI value is 8.49. The AmRk protein contains the conserved 7TM region (7 transmembrane receptor–rhodopsin family) and leucine repeats (LRRs) (S1E and S1F Fig), which is typical of G proteins-coupled receptors (GPCP), and serves as the ligand-binding site [34].

AmBurs α, AmBurs β and AmRk sequences were aligned with orthologue sequences of other arthropod species (S2 Fig). The presence of eleven cysteine residues in conserved positions, which is typical of CPK proteins, was evident in the Burs α and β subunits (S2A and S2B Fig). Phylogenetic analysis was performed to explore the evolutionary relationship among the honeybee bursicon subunits and their putative orthologues in other arthropod species. The phylogenetic trees were rooted with the Burs α or β subunit sequences of *Limulus polyphemus* (Chelicerata, Merostomata, Xiphosura). For both subunit trees, *Ixodes scapularis* (Chelicerata, Arachnida, Ixodida), sister group of Xiphosura, came closer to the root, and the Pancrustacea (‘Crustacea’ and Hexapoda) formed a group with a bootstrap support of 991 for Burs α and a low bootstrap support (459) for Burs β. The phylogenetic trees also supported a Vericrustacea clade, discriminating the Decapoda (Malacostraca), *Callinectes sapidus*, *Carcinus maenas*, *Homarus gammerus* and *Penaeus monodon*, from the water flea *Daphnia arenata* (Branchiopoda) with a bootstrap support value of 969 for Burs α and 809 for Burs β. The hymenopteran species were grouped separately from the lepidopteran and dipteran species. Among the dipterans, the flies, but not the mosquitoes (*Aedes aegypt* and *Anopheles gambie*) were clustered together in both trees (S3 Fig). As we obtained the same topology for both trees, we can suggest that the selection pressure for both subunits of bursicon was similar. Unfortunately, well-determined sequences of bursicon subunits are available only for a few taxa and this, at the moment, is a setback for more robust phylogenetic analyses. The low number of available sequences also may explain the position of the coleopteran *T*. *castaneum* close to a mosquito species in both trees.

*AmRk* shares similarities with orthologue sequences of other hymenopterans, dipterans and also a coleopteran and a lepidopteran species. The rickets sequences available in genomic data banks, however, are still insufficient to a well-supported phylogenetic analysis.

### Expression of *AmBurs α*, *AmBurs β* and *AmRk* depends on the ecdysteroid titer that triggers pupal cuticle apolysis and the onset of adult cuticle formation

**Fig 2:**
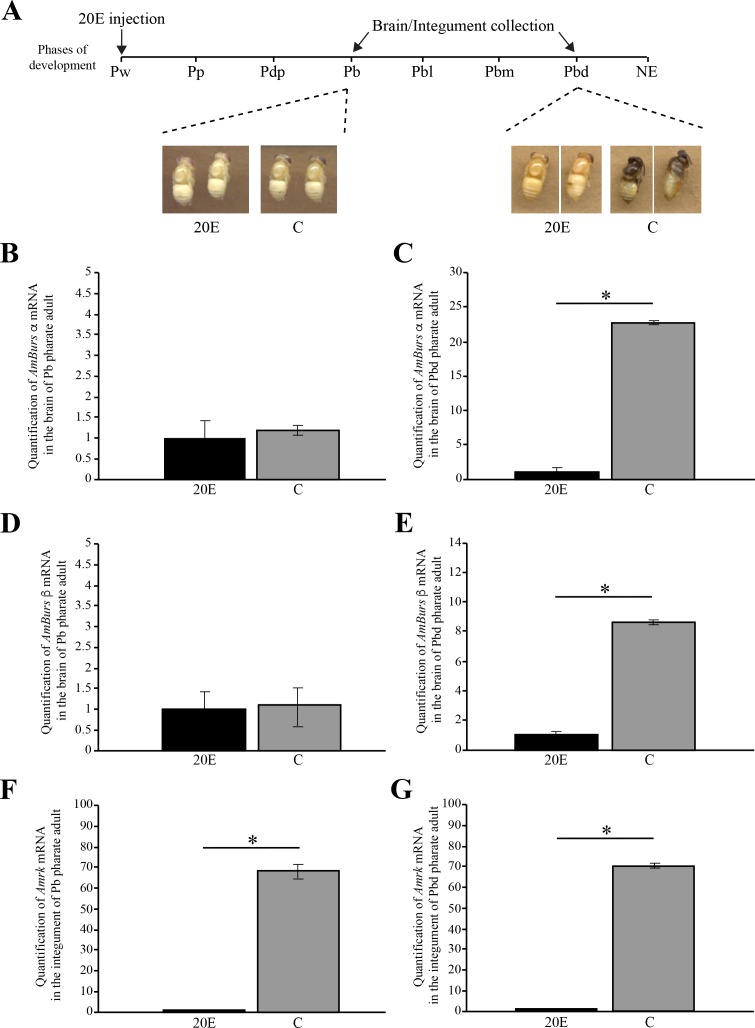
20E-dependent expression of *AmBurs α*, *AmBurs β* and *AmRk*. **(A)** Representation of the moment of 20E injection (Pw phase) and collection of brain and integument samples at the Pb and Pbd pharate adult phases. External aspect of the developing honeybees injected with 20E and (C) controls. *AmBurs α* transcript levels in the brain of **(B)** Pb and **(C)** Pbd phases. *AmBurs β* transcript levels in the brain of **(D)** Pb and **(E)** Pbd phases. *AmRk* transcript levels in the integument of **(F)** Pb and **(G)** Pbd phases. Transcript levels quantified through RT-qPCR. Bars represent mean ± se of three samples, each prepared with two brains or two integuments. The asterisks indicate statistically significant differences (Student's t-test, p<0,05).

As shown in Fig 1A, the concentration of circulating ecdysteroids increases during the pupal apolysis and then it decreases progressively in the pharate adult-phases, during which the adult cuticle is synthesized. By injecting Pw pupae with 20E, we prevented, or retarded, the drop in the levels of ecdysteroids. Hormone titer manipulation prevented the normal pigmentation and sclerotization, and also the correct formation, of the adult cuticle. This effect was more evident at the time corresponding to the end of the pharate adult stage (Pbd phase), as shown in the images of 20E-treated and respective control bees (Fig 2A). At the Pbl phase, brains of the 20E-injected bees displayed significantly lower levels of *AmBurs α* (Fig 2B and 2C) and *AmBurs β* (Fig 2D and 2E) transcripts than the respective controls injected with the 20E vehicle only, thus suggesting that the increase in the expression of bursicon genes in pharate-adults depends on the endogenous ecdysteroid titer decline. Similarly, the levels of *AmRk* transcripts were lower in the integument of 20E-injected bees (Fig 2F and 2G). In this case, and differently from bursicon, the significant effect of 20E could be observed earlier, at the Pb phase. These data thus indicate that the expression of bursicon receptor also depends on the ecdysteroid titer.

### Knockdown of *AmBurs α* and *AmBurs β* transcripts disturbs adult cuticle formation

**Fig 3:**
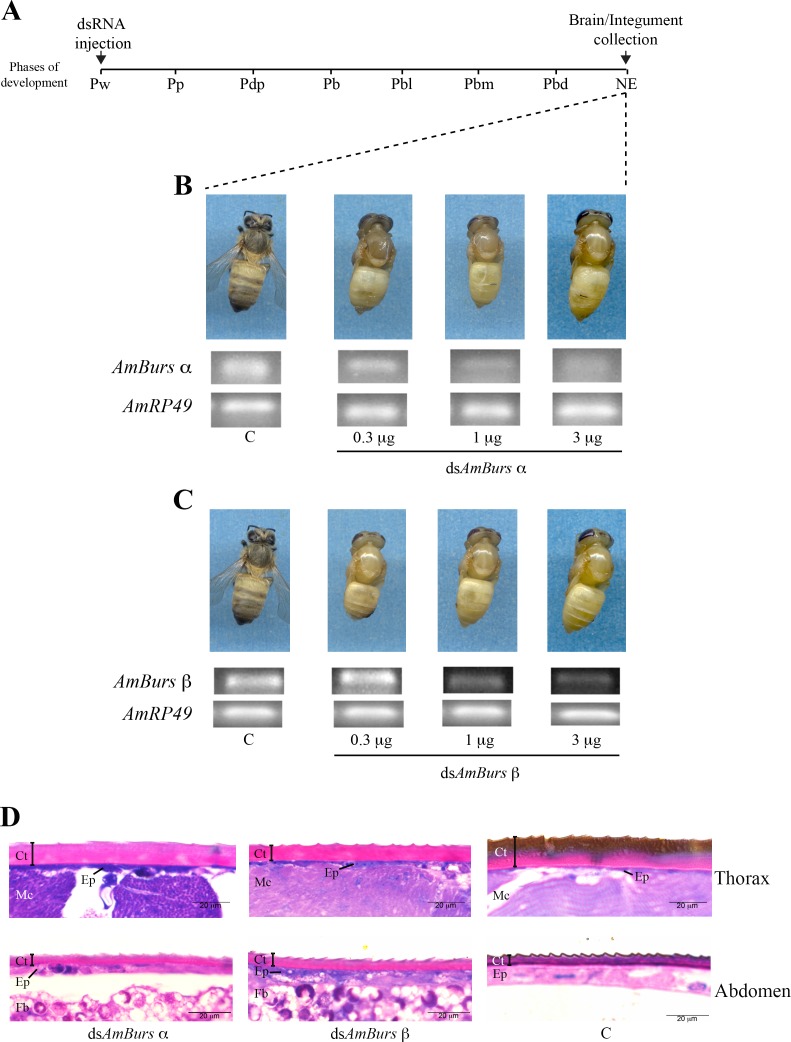
*AmBurs α* and *AmBurs β* knockdown mediated by dsRNA delivery into the abdominal cavity impairs the complete formation of adult cuticle, tanning and ecdysis. **(A)** Pupae (Pw) were injected with ds*AmBurs α* or ds*AmBurs β*; brain and integument samples were collected after ecdysis of the controls (NE phase). Bee phenotypes and brain transcript levels after injection of 0.3, 1 or 3 μg of **(B)** ds*AmBurs α* and **(C)** ds*AmBurs β* in comparison with untreated control, C. Transcript levels determined through RT-sqPCR using *AmRP49* as reference gene. **(D)** The thoracic and abdominal integuments were dissected from the dsRNA-treated and untreated bees and stained with methylene blue and basic fuchsin for microscopic examination. Cuticle (Ct), epidermis (Ep), thoracic musculature (Mc) and fat body (Fb) are identified in the histological sections. Note the melanized cuticle in the thoracic integument section of a control bee in contrast to the incompletely differentiated cuticle of the dsRNA-treated bees. The control abdominal cuticle shows a dark and toothed superficial layer, which was much less evident in the dsRNA-treated bees.

In order to investigate whether the expression of bursicon is important for the adult cuticle formation and tanning (melanization and sclerotization) during the pharate-adult stage, we interfered in the levels of *AmBurs α* and *AmBurs β* transcripts via injection of the specific double-stranded RNAs, ds*AmBurs α* or ds*AmBurs β*. The dsRNAs were injected into the abdomen before the onset of adult cuticle formation, in Pw pupae. Then, the pharate-adult development was followed up to the adult ecdysis of the control bees (Fig 3A), when the cuticular phenotype was recorded. Fig 3B and 3C shows the external aspect of bees representative of those groups of 15–20 ones that were treated with 0.3, 1 or 3 μg ds*AmBurs α* or ds*AmBurs β*. Both treatments impaired the normal progress of adult cuticle tanning and the ecdysis. Although not evident in the bees injected with ds*AmBurs β*, we also observed a subtle dose-dependent effect of the ds*AmBurs α*-treatment; bees injected with 0.3 μg showed the head and thoracic cuticle slightly more pigmented than those injected with 3 μg. Simultaneously, using RT-sqPCR, the levels of *AmBurs α* and *AmBurs β* transcripts were checked in the brains of dsRNA-treated bees and respective controls in order to confirm the effectiveness of the treatment. Transcript levels were lower in the treated bees, mainly in those treated with 1 or 3 μg dsRNA (Fig 3B and 3C). The thoracic and abdominal integuments of the dsRNA-treated and control bees were then prepared for histological sections, optical microscopy and image analysis. The thoracic cuticle of ds*AmBurs α-* and ds*AmBurs β*-treated bees did not show the melanized superficial layer typically seen in the control bees. Similarly, the abdominal cuticle did not differentiate correctly in the knocked out bees: the dark and toothed superficial cuticle layer seen in the control bees was not observed in the knocked out ones (Fig 3D).

The specificity of the effects of ds*AmBurs α* and ds*AmBurs β* was checked through the injection of a dsRNA against the gene encoding the storage protein hexamerin 70b (HEX 70b), highly expressed in the larval fat body [49]. We could observe that the bees injected with ds*AmHex70b* showed a normal phenotype and levels of *AmBurs α* and *AmBurs β* transcripts that were comparable to those observed in the non-injected controls (S4 Fig), thus confirming the specificity of the knockdown.

### Knockdown of *AmBurs α* and *AmBurs β* changes the expression of bursicon receptor (*AmRk*) and other genes expressed in the integument and involved in adult cuticle formation

**Fig 4:**
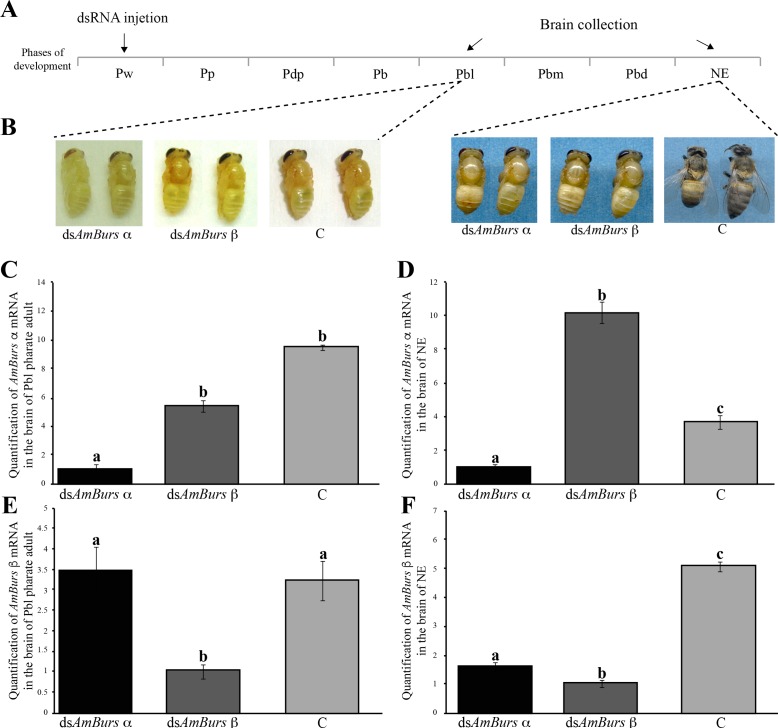
Effects of the *AmBurs α* and *AmBurs β* knockdown mediated by dsRNA delivery into the brain. **(A)** Pupae (Pw) were injected with ds*AmBurs α* or ds*AmBurs β*, and brain samples were collected when bees reached the Pbl pharate-adult phase and after ecdysis (NE phase). **(B)** At the Pbl phase, the effect of ds*AmBurs α* is slightly more pronounced than ds*AmBurs β* in arresting cuticle tanning. At the NE phase, the ds*AmBurs α-* and ds*AmBurs β*-treated bees showed a softer and very little pigmented cuticle than the control bees, C. **(C-F)** The specificity of *AmBurs α* and *AmBurs β* dsRNAs is revealed by the significantly different levels of these transcripts in brain samples of bees injected with **(C, D)** ds*AmBurs α* or **(E, F)** ds*AmBurs β* and collected at the **(C, E)** Pbl phase or at the **(D, F)** NE phase. Transcript levels determined through RT-qPCR using *AmRP49* as reference gene. Each bar represent mean ± se of four (n = 4) independent (biological) samples; each sample was prepared with a single brain. Different letters on the bars indicate statistically significant differences (Student's t-test, p<0,05).

So as to give more insight into the effects of bursicon knockdown, in further experiments we injected ds*AmBurs α* or *dsAmBurs β* directly into the brain of pupae (Pw phase) and chose two developmental points to quantify transcript levels and to examine the resulting cuticle phenotypes. The developmental points were the Pbl phase, when tanning is beginning in the adult cuticle [51], and the NE phase, which corresponds to the moment of adult ecdysis (Fig 4A). At the Pbl phase, we could observe a delay in the process of adult cuticle tanning (maturation), mainly in the bees injected with ds*AmBurs α*, in comparison to the controls. At the NE phase, both groups of bees, injected with ds*AmBurs α* or ds*AmBurs β*, showed arrested cuticle development and tanning (Fig 4B).

The levels of *AmBurs α* and *AmBurs β* transcripts in the brain were quantified in these bees using RT-qPCR. Levels of *AmBurs α* were significantly lower in the brains of bees injected with the specific dsRNA and collected at the Pbl or NE phases, than in bees injected with ds*AmBurs β* or non-injected controls (Fig 4C and 4D). Similarly, the levels of *AmBurs β* were significantly lower in the brains of bees injected with the specific dsRNA and collected at the Pbl or NE phases than in bees injected with ds*AmBurs α* or non-injected controls (Fig 4E and 4F). Three conclusions were drawn from these results: (1) transcript knockdown was specific; (2) *AmBurs β* transcript quantification in ds*AmBurs α*-treated bees, and *vice-versa*, served as adequate ‘controls’ for the dsRNA injection; and (3) similar results were obtained with intra-abdominal dsRNA injection (Fig 3) or dsRNA injection directly into the brain (Fig 4). The higher levels of *AmBurs α* transcripts in ds*AmBurs β-*treated bees than in non-injected controls (Fig 4D) may be explained if considering the tendency of bursicon transcript levels to decrease in a short interval after bee ecdysis (data not shown).

The effectiveness of the *AmBurs α* and *AmBurs β* knockdown mediated by dsRNA led us to investigate in the knocked out bees some selected genes expressed in the integument and involved in cuticle formation and tanning. We used RT-qPCR to quantify in the integument the transcript levels of six genes encoding structural cuticle proteins and three genes encoding enzymes involved in the melanization/sclerotization pathway, besides the *AmRk* bursicon receptor. In support to the RT-qPCR data, we searched for the expression of these genes in triplicates RNA-seq libraries prepared with integument from the Pbm and NE phases.

**Fig 5:**
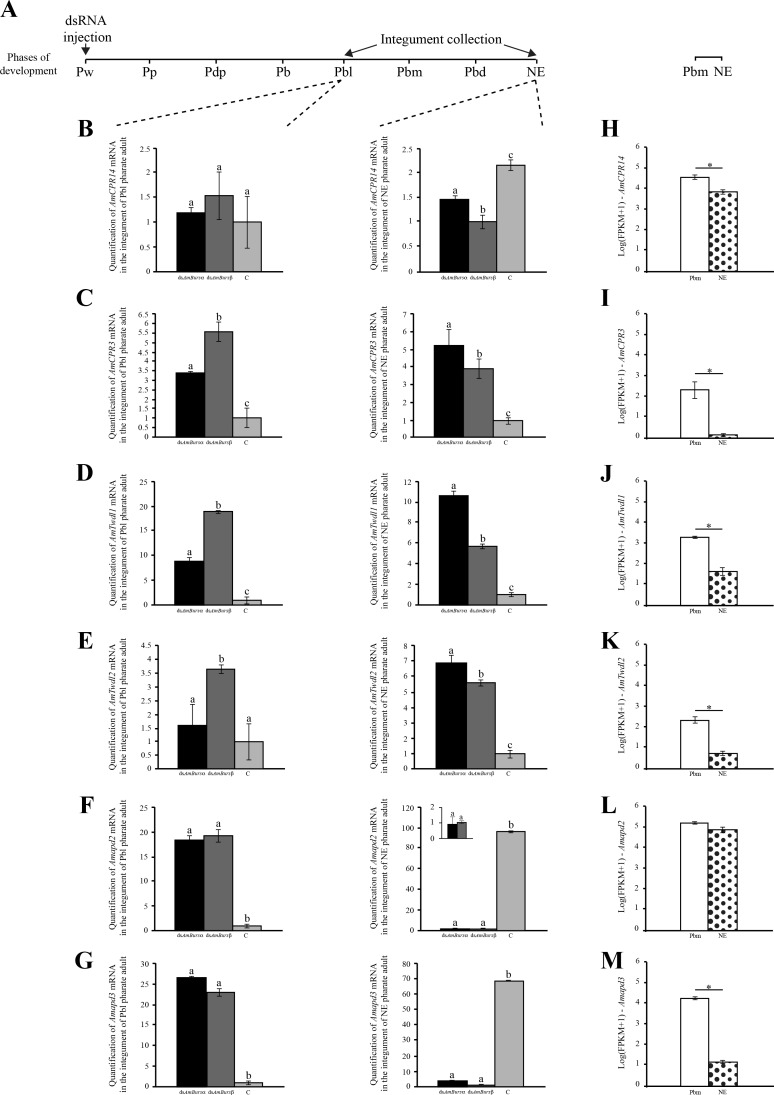
The expression of genes encoding structural cuticular proteins was up- or downregulated by *AmBurs α* and *AmBurs β* knockdown. **(A)** Pupae (Pw) were injected in the brain with ds*AmBurs α* or ds*AmBurs β*, and integument samples were collected when bees reached the Pbd and NE phases. Transcript levels of **(B)** *AmCPR14*, **(C)** *AmCPR3*, **(D)** *AmTwdl1*, **(E)** *AmTwdl2*, **(F)** *Amapd2* and **(G)** *Amapd3* were quantified in the integument through RT-qPCR using *AmRP49* as reference gene. Bars represent mean ± se of four samples, each prepared with a single integument. Different letters on the bars indicate statistically significant differences (Student's t-test, p<0,05). RNA-seq using integument samples support the presence of **(H)** *AmCPR14*, **(I)** *AmCPR3*, **(J)** *AmTwdl1*, **(K)** *AmTwdl2*, **(L)** *Amapd2* and **(M)** *Amapd3* transcripts during adult cuticle formation in pharate-adults (Pbm phase) and show that, except for *Amapd2* **(L)** (adjusted p-value = 0.226), there is a significant decrease in transcript levels after ecdysis (NE phase) (adjusted p-values ≤ 0.001).

The injection of ds*AmBurs α* or a ds*AmBurs β* into the pupal brain (Pw phase) affected the expression of genes encoding structural cuticular proteins. Pharate-adults (Pbl phase) and newly-emerged bees (NE phase) that developed from the dsRNA-injected pupae showed altered expression of *AmCPR14*, *AmCPR3*, *AmTwdl1*, *AmTwdl2*, *Amapd2* and *Amapd3* in the integument (Fig 5A). *AmCPR14* was downregulated at the NE phase (Fig 5B). The genes encoding AmCPR3, AmTwdl1 and AmTwdl2 proteins were upregulated. Increased transcript levels were clearly verified at the Pbl and NE phases for *AmCPR3* (Fig 5C) and *AmTwdl1* (Fig 5D), and at the NE phase for *AmTwdl2* (Fig 5E). ds*AmBurs β* caused the upregulation of *AmTwdl2* also at the earlier Pbl phase (Fig 5E). The apidermin genes, *Amapd2* and *Amapd3*, showed a specific profile pattern, in which both were upregulated at the Pbl phase, and this was followed by strong downregulation at the NE phase (Fig 5F and 5G). Therefore, the response of structural cuticular genes to bursicon transcript levels knockdown was evident, but not uniform. Fig 5H–5M shows the *status quo* of *AmCPR14*, *AmCRP3*, *AmTwdl1*, *AmTwdl2*, *Amapd2* and *Amapd3* transcript levels as detected in the RNA-seq libraries of integuments from the Pbm phase (which antecedes the Pbd phase; see Fig 1) and newly-ecdysed (NE) bees. Except for *Amapd2* (Fig 5l), which showed similar transcript levels in both phases, all the other genes were downregulated after the adult ecdysis (NE phase). Together, the data shown in Fig 5 indicate that by interfering in cuticle protein gene expression, bursicon influences adult cuticle formation. Furthermore, certain cuticle protein genes, as is the case of *Amapd2*, continue to be expressed in higher levels even after the ecdysis.

**Fig 6:**
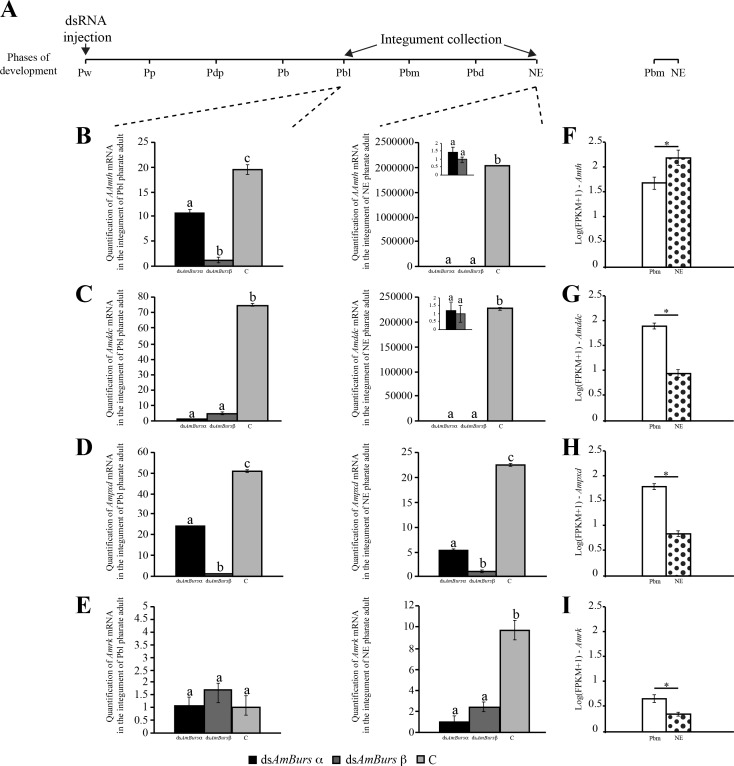
Effect of *AmBurs α* and *AmBurs β* knockdown on the expression of genes encoding enzymes with roles in the melanization/sclerotization pathway, and on the expression of the bursicon receptor. **(A)** Pupae (Pw) were injected in the brain with ds*AmBurs α* or ds*AmBurs β*, and integument samples were collected when bees reached the Pbl pharate-adult phase and after ecdysis (NE phase). Transcript levels of **(B)** *Amddc*, **(C)** *Amth*, **(D)** *Ampxd* and **(E**) *AmRk* were quantified in the integument through RT-qPCR using *AmRP49* as reference gene. Bars represent mean ± se of four samples, each prepared with the integument of a single bee. Different letters on the bars indicate statistically significant differences (Student's t-test, p<0,05). RNA-seq using integument samples support the presence of **(F)** *Amddc*, **(G)** *Amth*, **(H)** *Ampxd* and **(I)** *AmRk* transcripts during adult cuticle formation in pharate-adults (Pbm phase) and show that, except for *Amth* **(B)**, which was up-regulated, there is a significant decrease in transcript levels after ecdysis (NE phase) (adjusted p-values ≤ 0.001).

We also investigated the expression of three genes encoding enzymes involved in the melanization/sclerotization pathway, and the expression of the putative bursicon receptor, *AmRk*, in dsRNA*-*treated bees. As for the cuticular protein genes, the injection was ministered at the Pw phase, and transcripts were quantified through RT-qPCR at the Pbl and NE phases (Fig 6A). The levels of *Amth* (Fig 6B), *Amddc* (Fig 6C) and *Ampxd* transcripts (Fig 6D) were significantly downregulated in the integument of ds*AmBurs α-* and ds*AmBurs β-*treated bees, and this was verified at both Pbl and NE phases. These results highlighted that normal levels of bursicon expression are required for the pigmentation and sclerotization of the adult cuticle in pharate-adults. Similarly, the *AmRk* gene was also downregulated and this effect was observed at the NE phase (Fig 6E), possibly indicating a regulatory interaction between bursicon and its receptor at the transcriptional level. Fig 6F–6I shows the transcript levels of *Amth*, *Amddc*, *Ampxd and AmRk* genes as detected in the RNA-seq libraries prepared with integuments from the Pbm and NE phases (see Fig 1). Except for *Amth* transcript levels that increased significantly after the adult ecdysis (NE phases), the other three genes, *Amddc*, *Ampxd* and *AmRk* were all downregulated after ecdysis. The increase in *Amth* transcript levels after ecdysis suggests a corresponding increase in tyrosine hydroxylase synthesis and activity. If so, this result is intriguing considering that *Amddc*, which encodes the enzyme catalyzing the next reaction in the melanization/sclerotization pathway, was significantly downregulated after ecdysis, thus suggesting that cuticle tanning is coming to an end.

## Discussion

### Expression of bursicon genes related to cuticle formation and tanning during the pharate-adult stage

Previously, the honeybee bursicon hormone had been predicted as being the product of a single open reading frame representing the fusion of the α and β nucleotide sequences [30, 34]. This prediction was further revised, thus verifying that as in other arthropod species, there are two genes encoding bursicon subunits in the honeybee genome [52]. Here, the bursicon α and β coding sequences were characterized by sequencing almost entirely their corresponding cDNAs, thus validating the predictions in the honeybee genome assembly [53].

Transcripts for bursicon and its receptor were detected in pupae (Pw phase), pharate-adults (Pb, Pbl and Pbm phases) and newly-emerged (NE) adults of the honeybee, with increased levels in pharate-adults and NE adults. In *Drosophila*, the genes encoding bursicon and its receptor were expressed during all stages of development (larvae, prepupae, pupae, pharate-adults, adults), also with increased expression in pharate adults [29, 26]. The presence of transcripts for bursicon and its receptor during pharate-adult development was also reported for *T*. *castaneum* [54–55]. Such findings are consistent with roles of bursicon in adult cuticle formation, a critical event in the pharate-adult development. In support of this premise, the RNAi-mediated knockdown of the bursicon subunit genes in the honeybee impaired the complete thickening and tanning of the adult cuticle and, consequently, the ecdysis. A different experimental approach resulted in a comparable result in another arthropod species, the blue crab *Callinectes sapidus*. In this case, recombinant bursicon stimulated cuticle deposition and thickening in integument explants incubated *in vitro* [56], suggesting that such competence is evolutionarily conserved among arthropods. Similarly, the injection of dsRNA against the bursicon receptor in *T*. *castaneum* (*Tcrk*) final instar larvae, affected cuticle tanning and development of integumentary structures during the pupal stage, and caused a lethal arrest of the adult ecdysis [55]. However, this result is in contrast to the findings of a previous study, which differed in the time of the *Tcrk* dsRNA delivery. In this specific case, *Tcrk* dsRNA was injected in pharate-pupae and showed no effect on cuticle tanning [54]. Also, the tanning pattern was not altered in *B*. *mori* pupae submitted to bursicon transcripts knockdown via RNAi [57]. Thus, the time of treatment, or developmental stage that is chosen for the dsRNA delivery, seems preponderant in determining cuticular phenotypes. Distinct developmental events are going on during the larval and pupal stages, which may explain the different phenotypes. Also, insect species and developmental stages may differ in the intensity and duration of the response to RNAi, which may also interfere with the results.

The functions of bursicon and its receptor in inducing post-adult ecdysis events have been characterized in detail in *Drosophila* [20–22, 25, 26, 28, 58–60]. Apart from the regulation of cuticle tanning and wing expansion after the adult ecdysis, for which there is incontestable evidence, many questions remain regarding roles of bursicon at the ecdysis itself and in pharate stages, when a new cuticle is being synthesized. Bursicon is needed after the completion of ecdysis at each developmental stage to expand and harden the new cuticle [26]. It is required in combination with CCAP (crustacean cardio acceleratory peptide) and Mip (myoinhibiting peptide) for the pupal ecdysis of *Drosophila* [61]. The release of bursicon subunits was demonstrated in *Drosophila* at the pupal ecdysis. Experiments with *Drosophila* burs β-null mutants resulted in pharate-adult phenotypes that were consistent with deficiencies at pupal ecdysis [62]. Furthermore, to the bursicon receptor in *Drosophila*, it was ascribed roles in the puparium tanning and the hardening of the pharate-adult cuticle [63]. There is evidence that the release of bursicon in *Drosophila* larvae occurs in two waves, preceding and following the second larval ecdysis sequence. The first wave occurred before the release of the ecdysis-triggering hormone (ETH), the first hormone in the ecdysis-signaling cascade activated by the decrease in the ecdysteroid titer [63]. Therefore, bursicon roles seem not limited to the post-ecdysial period and ecdysis itself (ecdysial behavior). Our results herein reported for the honeybee are consistent with bursicon roles in adult cuticle formation and tanning during the pharate-adult stage. These results are supported by findings on the presence of bursicon transcripts in pharate-adults of *Drosophila* [29, 26] and *Tribolium* [54], on the deleterious effect of bursicon receptor-RNAi on integumentary structures development and adult ecdysis in this beetle [55], and by experiments showing that a recombinant bursicon stimulated cuticle deposition and thickening in integuments incubated in vitro [56].

Our results include circumstantial evidence that bursicon is targeting its receptor in the integument of pharate adults, where both would be critical for the adult cuticle development. Although RNAi is a valuable tool for functional studies, and consistently pointed to physiological roles of bursicon in the honeybee integument during the pharate-adult stage, it is still lacking the demonstration of the presence of active bursicon in the hemolymph or integument, which is the direct evidence, or concrete proof, of bursicon functionality at this stage. Further research on bursicon in the honeybee should focus on bursicon-positive neurons and terminals using specific antibodies for the two bursicon subunits in immunolocalization experiments associated with confocal microscopy. Target tissues and the hemolymph also should be used in Western blots with specific antibodies for the investigation of bursicon release at the pharate-adult stage.

### Bursicon dsRNAs interfered in the expression of genes encoding structural cuticle proteins and enzymes of the melanization/sclerotization pathway

Consistent with the altered cuticle phenotype induced by the dsRNA treatment against bursicon transcripts, we also observed significant changes in the expression of genes encoding structural cuticle proteins and enzymes of the melanization/sclerotization via. The knockdown of *AmBurs α* or *AmBurs β* transcripts caused a significant decrease in the expression of *Amddc*, *Amth* and *Ampxd*, thus indicating that bursicon positively regulates genes of the melanization/sclerotization pathway. The responses of the genes encoding structural cuticular proteins were not uniform, indicating that bursicon signalizes for the repression or induction of genes involved in cuticle formation. Interestingly, genes encoding the two members of the Tweedle protein family showed the same behavior following knockdown of bursicon gene expression. The same occurred with the Apidermin genes. However, the genes in the CPR family, *AmCPR14* and *AmCPR3*, showed contrasting behaviors. As considered previously [6], the classification of cuticular proteins (and, hence, cuticular genes) is exclusively based on defined domains, or motifs, such as the R&R chitin-binding Consensus typical of the CPR protein class. The regions outside this Consensus, however, differ among the CPR genes, which may imply in distinct regulatory properties, and could explain the contrasting behaviors of *AmCPR14* and *AmCPR3*.

A genome-wide microarray analysis using pupae, which developed from *Tcrk* dsRNA-treated larvae, led to the identification of 24 differentially expressed genes [55]. None of these genes were assigned as encoding cuticular proteins or enzymes of the tanning pathway. By analyzing the individual expression of candidate targets of bursicon in the integument of dsRNA-treated bees, we could demonstrate significant changes in gene expression related to adult cuticle formation and tanning. Our results are consistent with those from two other studies in *Drosophila*. A DNA microarray analysis of neck-ligated flies injected with recombinant bursicon revealed several upregulated genes, among them two genes encoding proteins bearing the chitin-binding peritrophin-A domain and the R&R Consensus, respectively [64]. Comparable results were reported also using neck-ligated flies injected with purified bursicon, followed by RNA-seq analysis. Among the genes surveyed in this analysis, there were up- and downregulated cuticular protein genes [65]. Differently from our experiments in which the dsRNA injection was performed before the onset of adult cuticle formation, the bursicon treatments in the flies were performed after the adult ecdysis. Therefore, independently of the developmental stage and experimental approach, these results in flies, along with ours, indicated that bursicon regulates the expression of cuticular protein genes.

Each bursicon target candidate showed a similar behavior in response to AmBurs α or AmBurs β knockdown, i.e., a gene induced by dsAmBurs α was also induced by dsAmBurs β. The same occurred in the case of gene repression. This was clearly observed when the analyses were performed with integuments from knocked out bees at the ecdysis time (NE phase) (see Figs 5B–5G and 6B–6E). Such similar response of the target genes is here interpreted as the heterodimer being required for cuticle formation and tanning. In *Drosophila*, Burs α and Burs β homodimers are not active in *in vivo* tanning assays and do not activate the Rk receptor [29]. Similarly, a recombinant bursicon heterodimer, but not the homodimer, was able to induce tanning in newly-ecdysed neck-ligated *Drosophila* [66].

Interestingly, the knockdown of bursicon also affected the expression of the gene *AmRk*. Assuming that *AmRk* encodes the bursicon receptor in the epidermal cells of *A*. *mellifera*, as its orthologue in *Drosophila* [28], the decrease in *AmRk* expression in bursicon-knockdown bees may configure a case of regulation by its ligand.

The RNA-seq data provided essential information on the transcript levels steady state of bursicon and candidate targets, before and after the adult ecdysis. Furthermore, RNA-seq libraries analyses validated the presence of *AmBurs α* and *AmBurs β* transcripts in the brain, as well as the expression of the potential targets in the integument, during adult cuticle formation. We found similar levels of *AmBurs α* and *AmBurs β* transcripts in the brain RNA-seq libraries of pharate-adults and newly-ecdysed bees, which was confirmed in the RT-qPCR analysis (Fig 1). However, except for *Amapd2* and *Amth*, the other genes potentially targeted by bursicon, including its receptor *AmRk*, showed significantly lower expression in the integument libraries of newly-ecdysed bees (NE) than in pharate-adults (Pbm phase). This is an expected result since the process of adult cuticle formation is coming to the end in the NE bees. The levels of *Amapd2* transcripts did not significantly change before and immediately after the adult ecdysis (NE phase), although a later decrease is a possibility. The levels of *Amth* were significantly increased immediately after the adult ecdysis. In *Drosophila*, bursicon induces the post-ecdysial tanning through the phosphorylation and activation of tyrosine hydroxylase. Therefore, post-ecdysial tanning regulation is on the translation and activity levels of this enzyme [31]. The strong downregulation of *Amth* transcripts in ds*AmBurs α* and ds*AmBurs β*-treated bees, which resulted in altered tanning phenotype, indicates that bursicon also exerts its action at the transcriptional level, and during the pharate-adult stage.

### The expression of bursicon and its receptor is conditioned by the ecdysteroid levels

The expression of bursicon and its receptor in the honeybee pharate-adults is dependent on the ecdysteroid titer decrease. By experimentally maintaining the levels of ecdysteroids high via a 20E injection, we significantly blocked the expression of *AmBurs α* and *AmBurs β* genes in the brain as well as the expression of *AmRk* in the integument. In consequence, the adult cuticle was not completely formed and tanned. That the expression of bursicon receptor is dependent on ecdysteroids was also demonstrated in wing tissues from *T*. *castaneum* pupae exposed *in vitro* to 20E [55].

The action of ecdysteroids is mediated by a heterodimeric protein complex formed by the ecdysone receptor (EcR) and its partner, ultraspiracle (Usp), and was originally described in *D*. *melanogaster*. Ecdysteroids induce the so-called early genes and the biosynthesis of regulatory proteins, which in turn activate effector, or late, genes in a secondary response [67]. Among the effector genes are those encoding cuticular proteins and enzymes in the tanning pathway, as verified in the lepidopteran *Spodoptera exigua* [68].

The decrease in the ecdysteroid titer after the apolysis-triggering peak signalizes for the onset of the adult cuticle synthesis, its deposition beneath the detached pupal cuticle, and tanning. The onset of cuticle tanning in the honeybee marks the pharate-adult Pbl phase [51]. The final pharate-adult phase, Pbd, is characterized by the strong pigmentation of the adult cuticle coating the head and thorax (see Fig 1). As we are herein showing, the expression of bursicon genes in pharate-adults, as well as of the gene encoding its receptor, is conditioned on the ecdysteroid titer decline. The ecdysteroid titer-dependent expression of bursicon and its receptor is consistent with roles in cuticle tanning during the pharate-adult stage.

In summary, our data showed that in the honeybee, the expression of the genes encoding bursicon and its receptor increases in pharate-adults, during the formation and tanning of the adult cuticle, and depends on decreasing levels of ecdysteroid molting hormones. A direct or indirect regulatory action of bursicon on genes involved in cuticle formation and tanning could be evidenced via knockdown of bursicon genes mediated by RNAi. The current study contributes to insights into new bursicon functions beyond the canonical ones related to cuticle tanning and wing expansion after the adult ecdysis. Our results with *A*. *mellifera* represent useful information for future comparative studies using representative species towards a more comprehensive understanding of the roles of bursicon in insect development.

## Supporting Information

S1 Fig Schematic representations of (**A, B**) *AmBurs α*, (**C, D**) *AmBurs β* and (**E, F**) *AmRk* genes and their respective deduced proteins. Exons and introns are indicated by boxes and lines, respectively, and the number of nucleotides is shown. The direction of transcription is from 5' to 3' end. The curved arrows localize the primers used for sequencing the genes and for quantifying expression levels. The presence of conserved domains in the deduced proteins is indicated.
(PDF)

S2 Fig Alignments of the amino acid sequences of bursicon subunits **(A)** Burs α and **(B)** Burs β, and **(C)** bursicon receptor, rickets, from different arthropod species. (*) fully conserved sites; (:) site conserved in one of the strong groups of samples; (.) site is conserved in one of the weak groups of samples. Species' name followed by an asterisk was used for rooting the phylogenetic trees. The graphs under the sequences indicate the level of conservation at each amino acid position. Species identification and sequence accession numbers are available in **S3 Table**.
(TIF)

S3 Fig Phylogenetic trees based on the amino acid sequences of the bursicon subunits, **(A)** Burs α and **(B)** Burs β, from different arthropod species. Vericrustacea (green); Hymenoptera (blue); Diptera, Brachycera flies (pink); Coleoptera: *Tribolium castaneum* (red branch); Diptera, Nematocera: *Aedes aegypty* or *Anopheles gambiae* mosquitos (pink). Chelicerata: *Limulus polyphemus*, *Metaseiulus occidentalis*, *Ixodes scapularis*. (*) taxon used for rooting the tree. The names of the species and the amino acid sequences' IDs are available in **S3 Table**. The phylogenetic trees were inferred by a Maximum Likelihood Analysis (MLA) with 1000 bootstrap replications. The scale bars indicate the number of amino acid substitutions.
(TIF)

S4 Fig Specificity of the effects of the dsRNAs against the *AmBurs α* and *AmBurs β* transcripts. Groups of pupae were injected with ds*AmBurs α*, ds*AmBurs β* or with a dsRNA designed to knockdown the gene encoding the storage protein hexamerin 70b (HEX 70b), which is highly expressed in the larval fat body. Another group was left untreated. The photos above show representative bees of the dsRNA-injected and non-injected controls (NTC) at the time of the adult ecdysis. Cuticle tanning and ecdysis were impaired in the ds*AmBurs α*- or ds*AmBurs β*-injected bees, but not in the ds*AmHex70b*-injected bees or non-injected controls. Transcript levels were determined through RT-sqPCR using *AmRP49* as reference gene, and were lower in bees injected with ds*AmBurs α* or ds*AmBurs β* (arrowheads).
(TIF)

S1 Table Identification, accession numbers and characteristics of the genes encoding bursicon, its receptor, structural cuticle proteins and enzymes of the melanization/sclerotization pathway. (XLS)

S2 Table Primer sequences used in the experiments. (DOCX)

S3 Table Accession numbers for the sequences of bursicon and its receptor of the different arthropod species used for alignment and phylogenetic tree reconstruction. (XLS)
