# Supplementary figures and images for: RNAi-Mediated Functional Analysis of Bursicon Genes Related to Adult Cuticle Formation and Tanning in the Honeybee, Apis mellifera
Source: PLoS One. 2016 Dec 1;11(12):e0167421. doi: 10.1371/journal.pone.0167421 (PMC5132263; doi:10.1371/journal.pone.0167421)

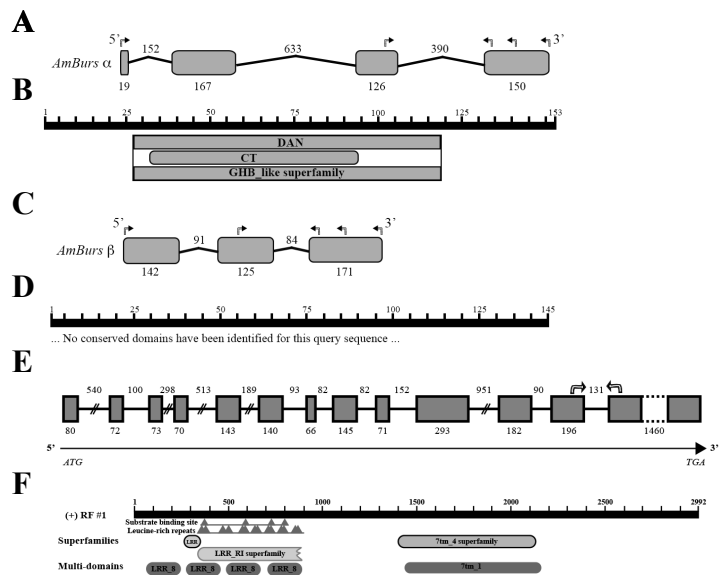

Supplement: S1 Fig — Schematic representations of (A, B) AmBurs α, (C, D) AmBurs β and (E, F) AmRk genes and their respective deduced proteins. Exons and introns are indicated by boxes and lines, respectively, and the number of nucleotides is shown. The direction of transcription is from 5' to 3' end. The curved arrows localize the primers used for sequencing the genes and for quantifying expression levels. The presence of conserved domains in the deduced proteins is indicated. (PDF) [file pone.0167421.s001.pdf]

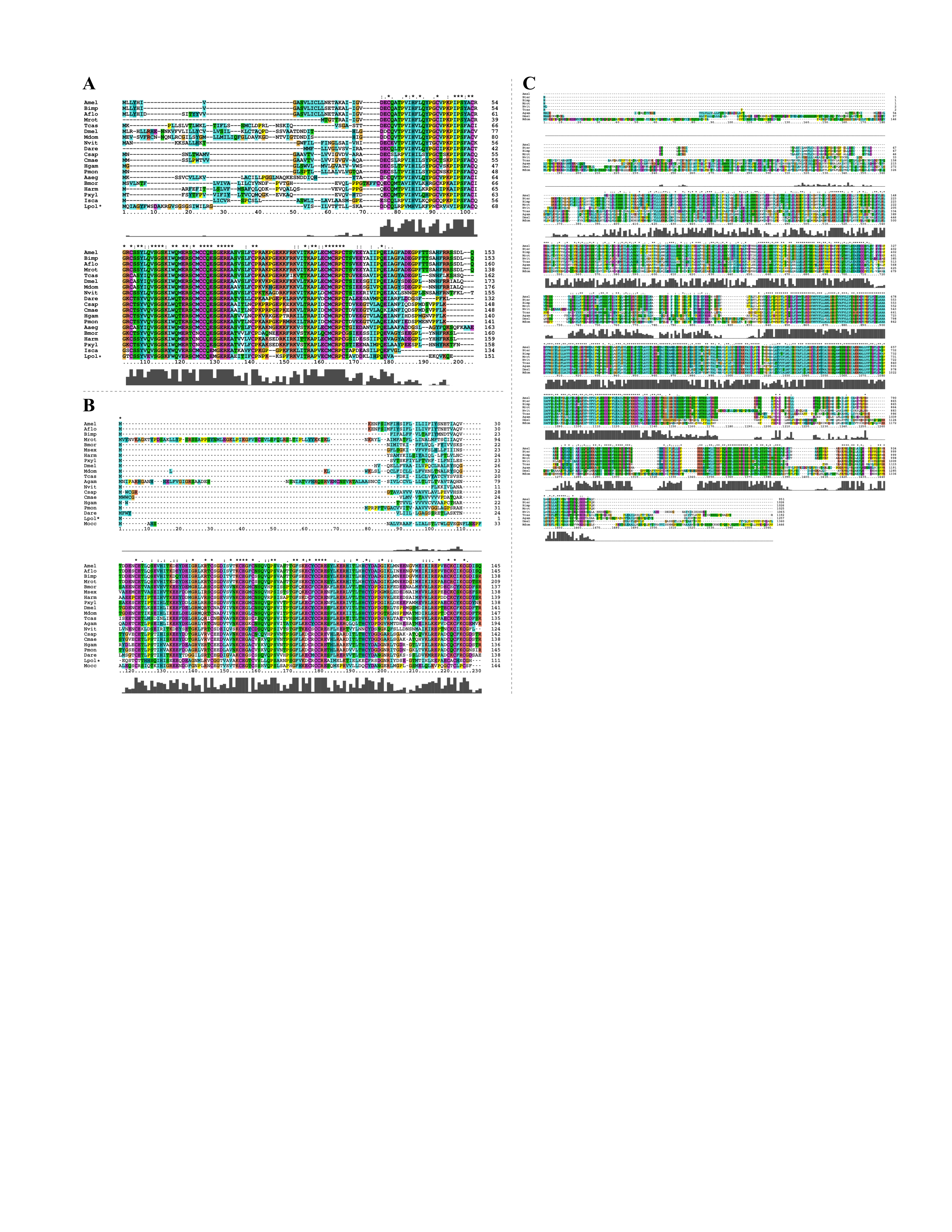

Supplement: S2 Fig — Alignments of the amino acid sequences of bursicon subunits (A) Burs α and (B) Burs β, and (C) bursicon receptor, rickets, from different arthropod species. (*) fully conserved sites; (:) site conserved in one of the strong groups of samples; (.) site is conserved in one of the weak groups of samples. Species' name followed by an asterisk was used for rooting the phylogenetic trees. The graphs under the sequences indicate the level of conservation at each amino acid position. Species identification and sequence accession numbers are available in S3 Table. (TIF) [file pone.0167421.s002.tif]

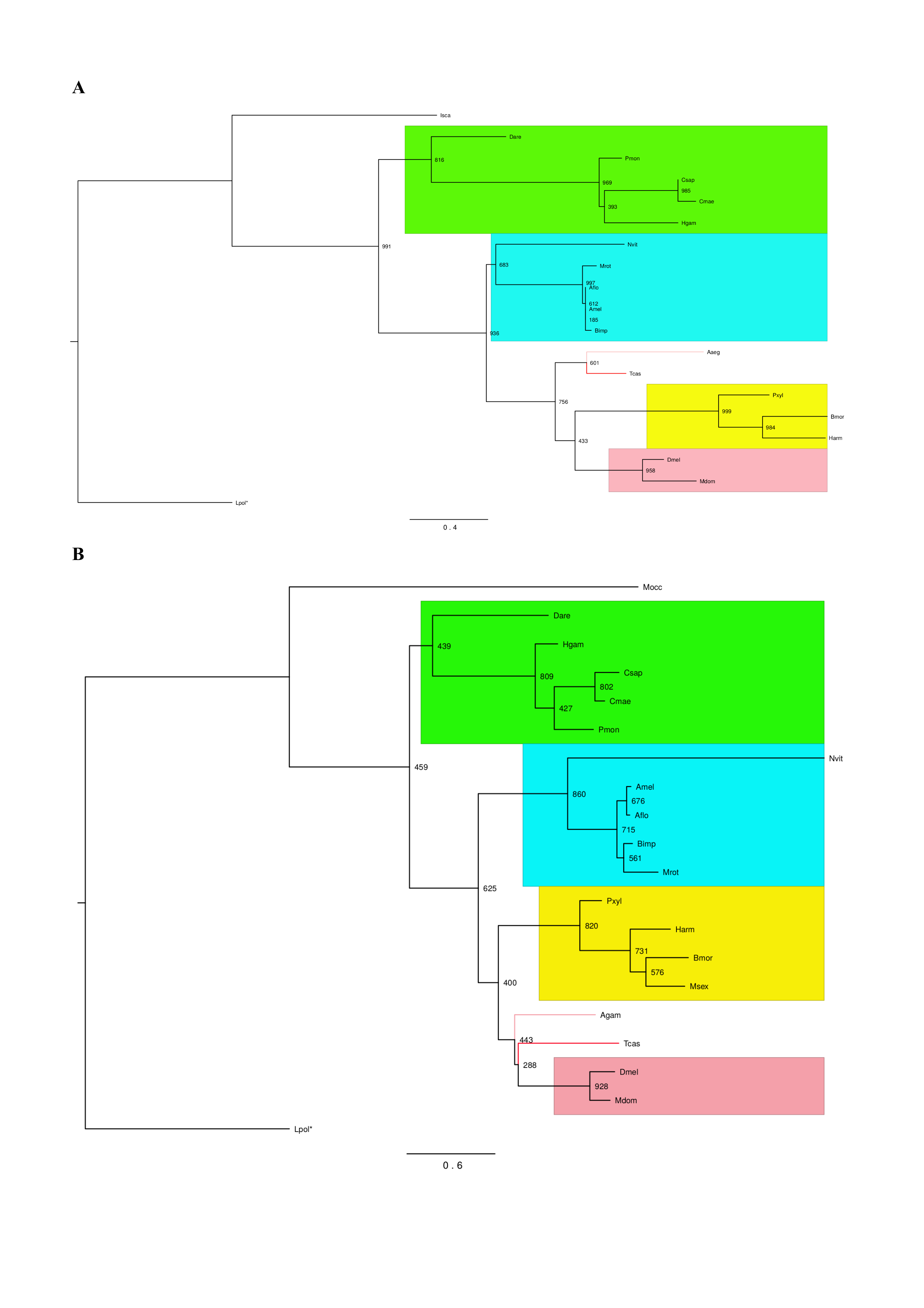

Supplement: S3 Fig — Phylogenetic trees based on the amino acid sequences of the bursicon subunits, (A) Burs α and (B) Burs β, from different arthropod species. Vericrustacea (green); Hymenoptera (blue); Diptera, Brachycera flies (pink); Coleoptera: Tribolium castaneum (red branch); Diptera, Nematocera: Aedes aegypty or Anopheles gambiae mosquitos (pink). Chelicerata: Limulus polyphemus, Metaseiulus occidentalis, Ixodes scapularis. (*) taxon used for rooting the tree. The names of the species and the amino acid sequences' IDs are available in S3 Table. The phylogenetic trees were inferred by a Maximum Likelihood Analysis (MLA) with 1000 bootstrap replications. The scale bars indicate the number of amino acid substitutions. (TIF) [file pone.0167421.s003.tif]

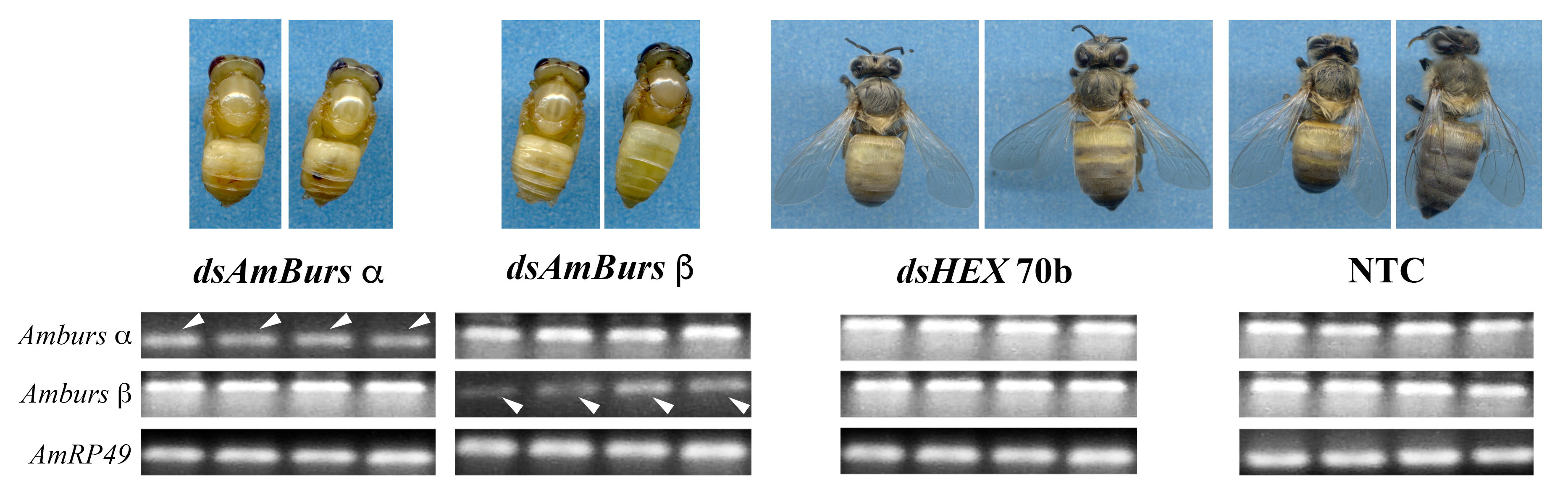

Supplement: S4 Fig — Groups of pupae were injected with dsAmBurs α, dsAmBurs β or with a dsRNA designed to knockdown the gene encoding the storage protein hexamerin 70b (HEX 70b), which is highly expressed in the larval fat body. Another group was left untreated. The photos above show representative bees of the dsRNA-injected and non-injected controls (NTC) at the time of the adult ecdysis. Cuticle tanning and ecdysis were impaired in the dsAmBurs α- or dsAmBurs β-injected bees, but not in the dsAmHex70b-injected bees or non-injected controls. Transcript levels were determined through RT-sqPCR using AmRP49 as reference gene, and were lower in bees injected with dsAmBurs α or dsAmBurs β (arrowheads). (TIF) [file pone.0167421.s004.tif]
