# Supplementary material for: RNAi-Mediated Functional Analysis of Bursicon Genes Related to Adult Cuticle Formation and Tanning in the Honeybee, Apis mellifera
Source: PLoS One. 2016 Dec 1;11(12):e0167421. doi: 10.1371/journal.pone.0167421 (PMC5132263; doi:10.1371/journal.pone.0167421)
Supplement: S2 Table — (DOCX) [file pone.0167421.s006.docx]

**S2 Table - Primer sequences used in the experiments. F and R: forward and reverse primers. The T7 promoter sequences are marked in bold and underlined.**

| **Experiment** | **Gene** | **Identification** | | | | **Primer name** | **Sequence** |
| --- | --- | --- | --- | --- | --- | --- | --- |
|  |  | **Version 4.0** | **Version 4.5** | | **Gene ID** |  |  |
| **Sequencing** | *Amburs a* | GB11959 | | GB45446 | 100049551 | *Amburs a* 1F Seq | **5’** GCG CAC AAT CTT TAA GCA CA **3’** |
|  |  |  |  |  |  | *Amburs a* 4R Seq | **5’** TTA TTG CAA ATC GGA AGA TCG **3’** |
|  |  |  |  |  |  | *Amburs a* 5R Seq | **5’** GTT ATG ATA CGA AGT TAG ATG **3’** |
|  | *Amburs β* | GB19117 | | GB45445 | 413045 | *Amburs β* 1F Seq | **5’** CAT GTT GGA GGG AAA GCT ACC **3’** |
|  |  |  |  |  |  | *Amburs β* 4R Seq | **5’** TTA TTG GGA TAT ATC ACC AC **3’** |
|  |  |  |  |  |  | *Amburs β* 5R Seq | **5’** ATA ATA ATT TTA TTA TCA CT **3’** |
|  |  |  | |  |  |  |  |
| **Sequencing &**  **Expression** | *Amburs a* | GB11959 | | GB45446 | 100049551 | *Amburs a* F | **5’** GCG AAA GAG AGG CCA GTG TA **3’** |
|  |  |  |  |  |  | *Amburs a* R | **5’** GGC AAA TCC AGC AAT CTC TT **3’** |
|  | *Amburs β* | GB19117 | | GB45445 | 413045 | *Amburs β* F | **5’** CAG GTA CAA CCA AGT GTA GCA A **3’** |
|  |  |  |  |  |  | *Amburs β* R | **5’** CAG GTA CAA CCA AGT GTA GCA A **3’** |
|  | *Amrk* | GB18043 | | GB18043 | 411738 | *Amrk F* | **5’** TCT TGT TTG CAA CCT TGC TG **3’** |
|  |  |  |  |  |  | *Amrk R* | **5’** ATG CGT TAT GGC GTA GTT CC **3’** |
|  |  |  | |  |  |  |  |
| **Knockdown** | *Amburs a* | GB11959 | | GB45446 | 100049551 | *dsBurs α F* | **5’ TAA TAC GAC TCA CTA TAG GGC GA**G GTG TTG ATG AAT GTC AAG CA **3’** |
|  |  |  |  |  |  | *dsBurs α R* | **5’ TAA TAC GAC TCA CTA TAG GGC GA**A CAC ATG CAG CTT CTT TCC A **3’** |
|  | *Amburs β* | GB19117 | | GB45445 | 413045 | *dsBurs β F* | **5’ TAA TAC GAC TCA CTA TAG GGC GA**T TGC TCA AGT AAC AGA TGA TGA GA **3’** |
|  |  |  |  |  |  | *dsBurs β R* | **5’ TAA TAC GAC TCA CTA TAG GGC GA**C AAA ATC CTT CAC ATT TTG TAA CAG **3’** |
| **Knockdown** | *AmHEX 70b* | GB10869 | | GB10869 | 406117 | *dsHEX 70b F* | **5’ TAA TAC GAC TCA CTA TAG GGC GA**C GAG TTC GTG GTT AAC CTG A **3’** |
|  |  |  |  |  |  | *dsHEX 70b R* | **5’ TAA TAC GAC TCA CTA TAG GGC GA**A CGA AGA CGG ATT CGT GGC T **3’** |
|  |  |  | |  |  |  |  |
| **Expression** | *Amddc* | GB13656 | | GB13656 | 410638 | *Amddc F* | **5’** gtg gtg agt cgc tag ctg aag **3’** |
|  |  |  |  |  |  | *Amddc R* | **5’** att cgc gac gac acc tat ct **3’** |
|  | *Amth* | GB15303 | | GB15303 | 408930 | *Amth F* | **5’** AGG GTT CAC GTT GAG ACC AG **3’** |
|  |  |  |  |  |  | *Amth R* | **5’** GCA TGT GTC CCA ACA GCT C **3’** |
|  | *Ampxd* | GU785071.2 | | GB19100 | 724541 | *Ampxd F* | **5’** AGA GTG AAC GAG CAG CTA GT **3’** |
|  |  |  |  |  |  | *Ampxd R* | **5’** ATA CAA CGT TTC ATC GTC CCA **3’** |
|  | *apd 2* | GB10737 | | GB10737 | 727161 | *apd 2 F* | **5’** CCT CTT GCA CCC ACT ATT GC **3’** |
|  |  |  |  |  |  | *apd 2 R* | **5’** CTG GGG CGG CTA CAA CTG **3’** |
|  | *apd 3* | GB30203 | | GB30203 | 100037419 | *apd 3 F* | **5’** GCT GGA CCA ACA CTA GTT GC **3’** |
|  |  |  |  |  |  | *apd 3 R* | **5’** TGG TGA GCG AGT ACA GAT GC **3’** |
|  | *AmelCPR 3* | GB17384 | | GB17384 | 409345 | *AmelCPR 3 F* | **5’** TTT GCG TAT GAC GTC CAA GA **3’** |
|  |  |  |  |  |  | *AmelCPR 3 R* | **5’** TAT GCG TCG AGT ACC GTC TG **3’** |
|  | *AmelCPR 14* | GB30336 | | GB30336 | 724777 | *AmelCPR 14 F* | **5’** CAA GCA ATG GGA TCA GCC AC **3’** |
|  |  |  |  |  |  | *AmelCPR 14 R* | **5’** GAA GCC ATT CTC GTC GGC TA **3’** |
|  | *AmelTwdl 1* | GB19234 | | GB19234 | 552281 | *AmelTwdl 1 F* | **5’** CAA ATC CAA GGA ACA GCA GC **3’** |
|  |  |  |  |  |  | *AmelTwdl 1 R* | **5’** GTG CTC CAT AAG AGG AGC TG **3’** |
|  | *AmelTwdl 2* | GB14193 | | GB14193 | 724398 | *AmelTwdl 2 F* | **5’** GTG GTT CTT CCC ACT CAA GC **3’** |
|  |  |  |  |  |  | *AmelTwdl 2 R* | **5’** TAT CCG AAG GAG GTT GTG CT **3’** |
|  | *Amrp 49* | GB10903 | | GB10903 | 406099 | *Amrp49 F* | **5’** CGT CAT ATG TTG CCA ACT GGT **3’** |
|  |  |  |  |  |  | *Amrp49 R* | **5’** TTG AGC ACG TTG AAC AAT GG **3’** |
